# In Vitro Effect of Acidic Solutions and Sodium Fluoride on Surface Roughness of Two Types of CAD-CAM Dental Ceramics

**DOI:** 10.1155/2021/9977993

**Published:** 2021-07-23

**Authors:** Elham Farhadi, Hamid Kermanshah, Shiva Rafizadeh, Reza Saeedi, Ladan Ranjbar Omrani

**Affiliations:** ^1^Dentistry Institute, Restorative Dentistry Department, School of Dentistry, Tehran University of Medical Sciences, Tehran, Iran; ^2^Dental Research Center, Dentistry Institute, Restorative Dentistry Department, School of Dentistry, Tehran University of Medical Sciences, Tehran, Iran; ^3^School of Dentistry, Bushehr University of Medical Sciences, Bushehr, Iran; ^4^School of Dentistry, Shahid Sadoughi University of Medical Sciences, Yazd, Iran

## Abstract

**Objectives:**

This study assessed the effect of immersion in acidic solutions and sodium fluoride on surface roughness of dental ceramics.

**Materials and Methods:**

40 blocks of Vitablocs Mark II and IPS e.max CAD (5 × 5 × 3) were prepared. The samples were divided into five groups (*n* = 8) for immersion in artificial saliva (control), artificially prepared gastric acid, acetic acid, 0.02% sodium fluoride + gastric acid, and 0.02% sodium fluoride + acetic acid. The samples were immersed for 168 hours in the respective solutions except for sodium fluoride, in which the samples were immersed for 69 hours. The surface roughness of samples was measured before and after immersion using a profilometer. The surface roughness changes of three specimens of each group were evaluated by scanning electron microscopy (SEM). Data were analyzed using one-way and two-way ANOVA, Tukey's test, and independent t-test.

**Results:**

Significant changes were noted in Ra (*P*=0.002) and Rq (*P* < 0.0001) in both types of ceramics. The lowest changes in Ra and Rq parameters were seen in artificial saliva and gastric acid and highest changes occurred following immersion in 0.02% sodium fluoride + acetic acid and 0.02% sodium fluoride + gastric acid, respectively. Changes in Rz were also significant following immersion in Vitablocs Mark II (*P* < 0.05). Immersion in 0.02% sodium fluoride + gastric acid and 0.02% sodium fluoride + acetic acid produced a rougher surface on both types of ceramics (SEM).

**Conclusion:**

Exposure of Vitablocs Mark II CAD and IPS e.max CAD to 0.02% sodium fluoride + gastric acid and 0.02% sodium fluoride + acetic acid significantly increased their surface roughness, while for Vitablocs Mark II, lager defects were seen on its surface.

## 1. Introduction

Dental erosion is an emerging dental problem that occurs due to exogenous or endogenous acidic factors in today's world [1]. The exogenous factors may include the consumption of acidic foods and beverages, while the endogenous factors may include exposure to gastric acid due to gastroesophageal reflux or recurrent vomiting [1, 2]. Gastric acid is very destructive to dental structures, and its content has a pH of 1 to 1.5, which is much lower than the critical pH for the dissolution of enamel [2].

Control of the factors creating dental erosion and preventive measures such as antacids and products containing fluoride, besides restoration of the affected teeth, is the treatment of choice for these patients [1]. Topical usage of fluoride results in the formation of a globular calcium fluoride-like layer; dissolution of this layer in acidic situations protects dental tissue against dissolution temporarily [3]. However, the effectiveness of fluoride in the prevention of acid erosion in restorative material is not clear.

Oral rehabilitation of patients with extensive erosive dental lesions is complex. Usually, a number of teeth are affected in these patients, and they often complain of poor esthetics and function [4]. Dental ceramics are among the commonly used dental materials for the fabrication of anterior and posterior restorations in these situations, due to optimal biocompatibility, excellent esthetics, and high wear resistance [5].

The stability of different ceramics in the oral environment is affected by their composition and microstructure, manufacturing techniques, pH of the environment, duration of exposure, and temperature of chemical agents [6, 7].

Exposure of ceramics to erosive materials causes their destruction due to leakage of alkaline ions, which eventually decreases their stability and flexural strength and increases the risk of crack formation and propagation [6]. Upon degradation of ceramics, their surface roughness increases, which enhances plaque accumulation wear of the opposing teeth or restorations, decreases their strength, increases discoloration, increases crack propagation, and negatively affects their clinical service [5, 7–11].

Fabrication of ceramic restorations by the computer-aided design/computer-aided manufacturing (CAD/CAM) technology is single-appointment and cost-effective. It enables the fabrication of restorations with excellent marginal and internal fit, lower technical sensitivity, and lower failure rate due to more homogenous ceramic structure and less defects [12].

Vitablocs Mark II CAD (Vita Zahnfabrik) is mainly composed of silicon dioxide (silica or quartz) with variable percentages of alumina. It has been reported that this dental ceramic has the highest resistance to abrasion due to the small size of particles (averagely 4 *µ*m) and its sintering process [6]. IPS e.max CAD (Ivoclar Vivadent) is a lithium disilicate glass ceramic designed for the computer-aided design/computer-aided manufacturing (CAD/CAM) technique. It has high strength and optimal marginal integrity. Etching of this ceramic solubilizes its glass phase [6].

Dental ceramics are constantly exposed to acidic agents and fluoride (in toothpastes and mouthwashes) in the oral environment. On the other hand, studies on the effects of acidic environments (especially gastric acid) and mouthwashes on surface roughness of different types of ceramics are limited. Thus, this study aimed to assess the effect of acidic environment and sodium fluoride on surface roughness of two commonly used dental ceramics.

## 2. Materials and Methods

This in vitro, experimental study evaluated two etchable dental ceramics, namely, Vitablocs Mark II CAD and IPS e.max CAD. Sample size was calculated to be a minimum of 8 ceramic blocks in each group according to a previous study assuming alpha = 0.05, beta = 0.2, and study power of 80% using one-way ANOVA power analysis feature of PASS [13].

### 2.1. Sample Preparation

After mounting of Vitablocs Mark II CAD and IPS e.max CAD (Ivoclar Vivadent, Schaan, Liechtenstein) blocks, ceramic blocks measuring 5 mm in length, 5 mm in width, and 3 mm in height were sectioned using a diamond-coated cutting disc (Mecatome T201A, Presi, Grenoble, France) under copious water irrigation. The accuracy of the samples was checked by an electron caliper for 0.1 mm (Mitutoyo Co, Kanagawa, Japan).

Crystallization of IPS e.max CAD samples was done by sintering at 850°C for 30 min, in an electric furnace (Programator P300, Ivoclar Vivadent, Liechtenstein).

One surface of the samples was polished using 400-, 600-, 800-, 1000-, 1200-, and 1500-grit abrasive papers, in a polishing machine (Malek Teb, Iran) under copious water irrigation.

A total of 40 cubic blocks were made of each ceramic type.

Next, the Vitablocs Mark II and e-max ceramic blocks were randomly divided into five groups (*n* = 8).

### 2.2. Preparation of Solutions

- Artificial gastric acid was prepared by dissolving 2.0 g sodium chloride and 3.2 g pepsin in 7.0 mL hydrochloric acid and water to reach a final volume of 1000 mL. Its pH was adjusted to 1.14.
- 4% acetic acid was prepared by diluting 100% acetic acid with a pH of 2.45.
- Artificial saliva was prepared using a combination of 1 M NaCl, 0.2 M NaH_2_PO_4_, 1 M acetic acid, 0.2 M CaCl_2_, and 2.0% NaN_3_ at a pH of 6.3.
- FluoriGard (Colgate-Palmolive) 0.02% sodium fluoride mouthwash was prepared fresh daily.

### 2.3. Immersion in Solutions and Measurement of Surface Roughness

Prior to immersion, the surface roughness parameters (Ra, Rz, and Rq) of all samples were measured and recorded in micrometers (*µ*m) as the baseline surface roughness. For this purpose, a laser scanning profilometer (TR200; Time Group; USA) was used at three different points on the surface of each sample.

Next, each block was immersed in a capped test tube containing 20 mL of the respective solution, namely, artificial saliva (control), artificially prepared gastric acid, acetic acid, 0.02% sodium fluoride + gastric acid, and 0.02% sodium fluoride + acetic acid. All samples, except for those immersed in sodium fluoride, remained in the respective solutions for 168 hours, corresponding to 90 seconds of exposure to gastric acid per day for 22 years. This immersion time also corresponded to clinical service of a metal-ceramic restoration in the oral cavity.

In brief, the following five groups were evaluated:

 
*Group 1*. Samples were immersed in artificial gastric acid (HCl) and incubated at 37°C for 168 hours
   *Group 2*. Samples were immersed in FluoriGard (0.02% sodium fluoride) and incubated at 37°C for 69 hours (corresponding to daily rinsing of sodium fluoride for 30 seconds), and the solution was changed after 24 hours and then immersed in artificial gastric acid (HCl) and incubated at 37°C for 168 hours
 
*Group 3*. Samples were immersed in acetic acid and were placed in an oven at 80°C for 168 hours
   *Group 4*. Samples were immersed in FluoriGard (0.02% sodium fluoride) and incubated at 37°C for 69 hours, the solution was changed after 24 hours and then were immersed in acetic acid and placed in an oven at 80°C
   *Group 5*. Samples were immersed in artificial saliva and incubated at 37°C for 168 hours (negative control)

After immersion, the samples were rinsed with distilled water and air-dried. The surface roughness of samples was then measured again.

To assess the effect of different solutions on the morphology of ceramic surface in microscopic scale, one specimen of each group was selected and mounted on aluminum stub and sputter coated with gold/palladium coated for evaluation under field emission SEM at x500 magnification (S-4160; Hitachi, Tokyo, Japan).

### 2.4. Statistical Analysis

Two-way ANOVA was used to assess the effect of immersion solution, type of ceramic, and their interaction on changes of Ra, Rq, and Rz surface roughness parameters. One-way ANOVA was used to assess the effect of type of ceramic on changes of roughness parameters. Pairwise comparisons were performed using Tukey's test. Student's t-test was used to analyze the changes in surface roughness after the intervention in different solutions for each of the two types of ceramics. Statistical analyses were carried out using SPSS version 22 (SPSS Inc., IL, USA) and *P* < 0.05 was considered statistically significant.

## 3. Results

The mean and standard deviation of Ra, Rq, and Rz parameters before and after immersion in different solutions of two types of ceramics were measured. By subtracting the Ra, Rq, and Rz parameters after immersion in the solutions from the corresponding baseline values, the magnitude of change in these parameters was determined (Tables 1 and 2).

One-way ANOVA showed significant differences regarding changes in Ra (*P*=0.002) and Rq (*P* < 0.0001) parameters after the immersion in the solutions. However, there was no significant difference in this regard between the two types of ceramics.

With regard to Ra, the smallest change was noted after immersion in artificial saliva, followed by gastric acid, acetic acid, sodium fluoride + acetic acid, and sodium fluoride + gastric acid. There was significant difference between groups immersed in saliva and gastric acid with groups immersred sodium fluoride + acetic acid and sodium fluoride + gastric acid (Table 3).

With regard to Rq, the smallest change was noted after immersion in artificial saliva followed by gastric acid, acetic acid, sodium fluoride + acetic acid, and sodium fluoride + gastric acid (Table 4).

Student's t-test showed significant differences with regard to changes in Rq parameter following immersion in saliva and gastric acid with following immersion in sodium fluoride + acetic acid and sodium fluoride + gastric acid.

According to two-way ANOVA, significant differences were noted in Rz parameter of Vitablocs Mark II CAD following immersion in different solutions (*P* < 0.0001) but these changes were not significant for e.max CAD (*P*=0.26).

Table 5 shows the results of pairwise comparisons of changes of Rz parameter of Vitablocs Mark II CAD. The lowest change in Rz parameter of Vitablocs Mark II CAD was noted following immersion in artificial saliva followed by gastric acid, acetic acid, sodium fluoride + acetic acid, and sodium fluoride + gastric acid. There was a significant difference following immersion in sodium fluoride + acetic acid and sodium fluoride + gastric acid with immersion in other solutions.

**Figure 1:**
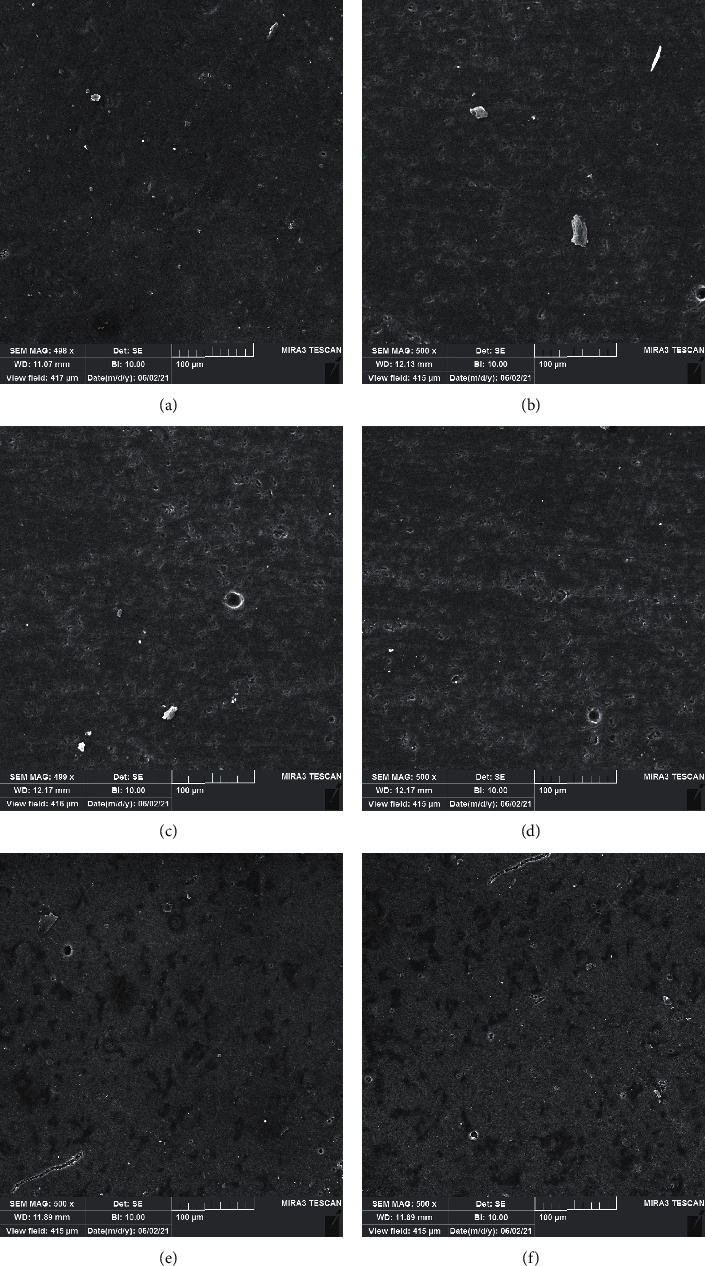
Scanning electron microscope photomicrographs of IPS e.max CAD (a) before immersion and after immersion in (b) saliva, (c) acetic acid, (d) gastric acid, (e) fluoride and acetic acid, and (f) fluoride and gastric acid 4 (500X magnification).

**Figure 2:**
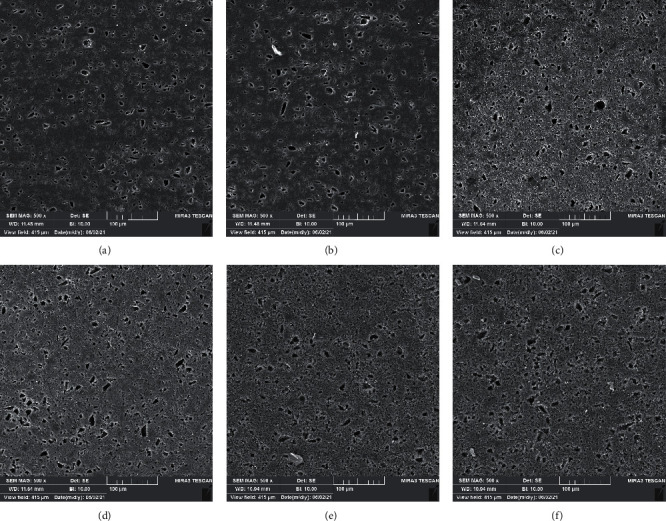
Scanning electron microscope photomicrographs of Vitablocs Mark II (a) before immersion and after immersion in (b) saliva, (c) acetic acid, (d) gastric acid, (e) fluoride and acetic acid, and (f) fluoride and gastric acid 4 (500X magnification).

Figures 1 and 2 show SEM evaluation of SEM of the e.max CAD ceramic and Vitablocs Mark II CAD, before immersion and after immersion in different solutions. Before immersion, the ceramics showed typically smoother surface. After immersion in different solutions and even saliva, there were surface changes. The highest surface roughness was seen in groups immersed in sodium fluoride + gastric acid and sodium fluoride + acetic acid.

## 4. Discussion

The effects of immersion in different acidic solutions on surface roughness of two types of ceramics, namely, Vitablocs Mark II CAD feldspathic ceramic and IPS e.max CAD, were evaluated in this study. Previous studies have reported the clinical service of metal-ceramic restorations in the oral environment to be around 20 years [14, 15]. In this study, the immersion time was 168 hours in acidic solutions and 69 hours in FluoriGard corresponding to 22 years of clinical service, in order to assess the long-term effects of acidic solutions [7]. The samples were polished by silicon carbide disc, since evidence shows that it is an acceptable method of polishing [16].

In this study, three surface roughness parameters were evaluated, namely, Ra (average surface roughness), Rq (root mean square roughness), and Rz (sum of the largest profile peak and the largest profile valley). Ra provides two-dimensional data about the surface evaluated, while Rq is the square root of the spreading of surface elevation and provides more precise three-dimensional data. It is also more sensitive to the peaks and depressed areas of the evaluated surface [17–19]. Rz is the difference in height among the average of five highest peaks and five lowest valleys and reveals the presence of deep valleys in the surface of tested groups [20].

The results of changes in roughness parameters, and SEM images, showed that immersion of both types of CAD ceramics in acidic solutions increased their surface roughness. Regarding the changes in Ra parameter, immersion in artificial saliva caused the lowest and immersion in sodium fluoride + gastric acid caused the highest change in this parameter; the change in Ra parameter between saliva and sodium fluoride + gastric acid and sodium fluoride + acetic acid was significant in both types of ceramics. The same was true for changes in Rq, while there was a significant difference in changes in Rq between saliva, and gastric acid with sodium fluoride + gastric acid and sodium fluoride + acetic acid solution in both type of ceramics.

Vitablocs Mark II CAD and IPS e.max CAD are glass ceramics, which are susceptible to acid dissolution. Dissolution of glass particles leads to formation of surface defects in the ceramics [20]. Two mechanisms are involved in the process of ceramic degradation: selective leakage of alkaline ions and dissolution of ceramic silicate network (Si-O-Si). These mechanisms involve penetration of hydrogen ions or hydronium ions from the aqueous environment into the ceramic and release of alkaline ions from the ceramic surface into the aqueous solution to preserve their electric property. These observations have been previously confirmed by scanning electron microscopy as well [13, 21]. Release of silicone ions and other ions such as potassium and sodium can cause porosities in the glass matrix [22].

Kukiattrakoon et al. [13] reported that immersion of ceramics in acetic acid significantly increased their surface roughness, which was not in line with our findings. Acetic acid is a weak organic acid, which is used for chemical stability testing [23]. It has a chelating effect and might be corrosive for ceramics [22]. Such differences in the results may be related to differences in evaluated ceramics, since the CAD/CAM ceramics have less flaws, and more homogenous structure [24].

Gastric acid might also dissolve ceramics with glass matrix due to its low pH [25]. Some other studies showed that surface roughness increased by immersion in gastric acid, which was not in line with our findings [11, 25–27]. This might be due to the difference in the gastric acid prepared since they did not add proteolytic pepsin enzyme to their gastric acid solution. The results are controversial regarding the erosive effect of adding pepsin to HCl on the tooth structure, and there is no study on the effect of HCl + pepsin on restorative materials [28, 29].

In both ceramics, significant change in surface roughness was seen following immersion in sodium fluoride + acetic acid, and sodium fluoride + gastric acid. Use of mouthwashes containing NaF especially with an acidic pH creates hydrofluoric acid. Resultantly, the ceramic surface is subjected to high amounts of F^−^ that react with Si, Na, and K ions and create NaF, KF, and SiF_4_. These products are dissolved by acids [30–32].

Significant changes were noted in the Rz parameter of Vitablocs Mark II following immersion in different acidic solutions, which shows that on the surface of this ceramic large defects have been created. However, these changes were not significant for IPS e.max CAD. The lowest and the highest changes in Rz of Vitablocs Mark II CAD were reported following immersion in saliva and sodium fluoride + gastric acid, respectively.

The difference between Rz parameter of two types of ceramics might be related to difference in molecular distribution and composition, the specific internal structure, microstructure, different grain size, presence of different oxides, homogeneity, and impurities [27, 33].

In terms of microstructure, IPS e.max CAD ceramic is a lithium disilicate glass, with about 70% crystal and 1.5 *µ*m grain size in a glassy matrix composed of silica, lithium metasilicate, disilicate, and phosphate crystals [33, 34]. Its lithium-containing glass phase is a ternary phase of Li-Si-K-O with zirconium oxides that are more stable to acid corrosion, and large defects will not create on its surface [27, 30, 35].

Vitablocs Mark II ceramic has a fine feldspar structure with 4 *µ*m irregular crystalline phases such as insoluble feldspars, leucite crystals, and alumina particles in a weak feldspathic glass matrix [34, 36]. Due to its heterogeneous microstructure, and many micropores and channels on the surface, this ceramic degrades inconsistently, which increases the Rz surface roughness parameter [33].

A question may arise regarding the use of surface roughness parameter as a method to assess surface degradation. It should be noted that it is a time-dependent process and according to the exposure time and the environment, surface roughness may decrease [13]. Thus, higher surface roughness of samples subjected to sodium fluoride + gastric acid and sodium fluoride + acetic acid does not necessarily translate to higher surface degradation but may indicate gradual and continuous corrosion given that the samples are exposed to the respective solution for a long period of time.

It should be noted that our study had an in vitro design. In vitro conditions are different from the clinical setting. In the oral environment, the acidity of foods and drinks or fluoride compounds is neutralized by the buffering capacity of the saliva. Also, the oral environment is complex due to the presence of saliva, thermal alterations, and pH changes, which can differently affect the restoration properties [6]. Thus, the effects of acidic solutions and fluoride on ceramic restorations should be evaluated in the oral environment. Future studies are required to assess the effect of other acidic solutions on other types of restorations such as zirconia ceramics in vitro and in the clinical setting to further elucidate this topic.

## 5. Conclusion

Exposure of Vitablocs Mark II and IPS e.max CAD to acidic solutions and sodium fluoride significantly increases their surface roughness, while in E.max CAD large defects were not detected. Thus, it should be kept in mind that preventive measures involving the use of fluoride should not be preferably used in patients with erosive lesions.

## Figures and Tables

**Table 1:** Mean and standard deviation of changes of Ra, Rq, and Rz parameters of Vitablocs Mark II CAD before and after immersion in different solutions.

Solution	ΔRa	ΔRq	ΔRz
Saliva	−0.03 (0.17)	−0.04 (0.2)	−0.38 (0.62)
Acetic acid	0.25 (0.25)	0.32 (0.28)	1.37 (0.66)
Gastric acid	0.07 (0.22)	0.09 (0.31)	0.18 (1.31)
Sodium fluoride + acetic acid	0.42 (0.22)	0.54 (0.26)	2.88 (0.93)
Sodium fluoride + gastric acid	0.43 (0.21)	0.57 (0.26)	3.08 (1.21)

**Table 2:** Mean and standard deviation of changes in Ra, Rq, and Rz parameters of IPS e.max CAD before and after immersion in different solutions.

Solution	ΔRa	ΔRq	ΔRz
Saliva	0.13 (0.20)	0.17 (0.24)	0.71 (0.72)
Acetic acid	0.1 (0.19)	0.13 (0.23)	0.27 (0.72)
Gastric acid	0.09 (0.18)	0.1 (0.23)	0.4 (1)
Sodium fluoride + acetic acid	0.23 (0.4)	0.29 (0.45)	1.05 (1.23)
Sodium fluoride + gastric acid	0.26 (0.29)	0.32 (0.37)	1.28 (1.32)

**Table 3:** Pairwise comparisons of different solutions regarding the changes in Ra after immersion using Tukey's test.

Solution 1	Solution 2	Mean difference	Std. error	*P* value
Gastric acid	Saliva	0.032	0.087	0.99
	Acetic acid	0.092	0.087	0.83
	Sodium fluoride + acetic acid	0.242	0.087	0.05
	Sodium fluoride + gastric acid	0.26	0.087	0.03

Acetic acid	Saliva	0.124	0.087	0.61
	Sodium fluoride + gastric acid	0.168	0.087	0.31
	Sodium fluoride + acetic acid	0.149	0.87	0.43

Sodium fluoride + gastric acid	Saliva	0.292	0.087	0.01
	Sodium fluoride + acetic acid	0.018	0.087	1.0

Sodium fluoride + acetic acid	Saliva	0.274	0.087	0.02

**Table 4:** Pairwise comparisons of different solutions regarding the changes in Rq after immersion using Tukey's test.

Solution 1	Solution 2	Mean difference	Std. error	*P* value
Gastric acid	Saliva	0.043	0.107	0.99
	Acetic acid	0.129	0.107	0.75
	Sodium fluoride + acetic acid	0.32	0.107	0.03
	Sodium fluoride + gastric acid	0.352	0.107	0.01

Acetic acid	Saliva	0.171	0.107	0.8
	Sodium fluoride + gastric acid	0.224	0.107	0.24
	Sodium fluoride + acetic acid	0.192	0.107	0.39

Sodium fluoride + gastric acid	Saliva	0.395	0.107	0.004
	Sodium fluoride + acetic acid	0.032	0.107	0.99

Sodium fluoride + acetic acid	Saliva	0.363	0.107	0.01

**Table 5:** Pairwise comparisons of changes in Rz parameter of Vitablocs Mark II after immersion.

Solution 1	Solution 2	Mean difference	Std. error	*P* value
Gastric acid	Saliva	0.559	0.493	0.79
	Acetic acid	1.19	0.493	0.14
	Sodium fluoride + acetic acid	2.7	0.493	0.0001
	Sodium fluoride + gastric acid	2.9	0.493	0.0001

Acetic acid	Saliva	1.75	0.493	0.009
	Sodium fluoride + gastric acid	1.51	0.493	0.01
	Sodium fluoride + acetic acid	1.71	0.493	0.03

Sodium fluoride + gastric acid	Saliva	3.459	0.493	0.001
	Sodium fluoride + acetic acid	0.199	0.493	0.99

Sodium fluoride + acetic acid	Saliva	3.26	0.493	0.001

## Data Availability

The data used to support the findings of this study are available from the corresponding author upon request.
